# Regeneration of Exhausted Palladium-Based Membranes: Recycling Process and Economics

**DOI:** 10.3390/membranes12070723

**Published:** 2022-07-21

**Authors:** Luigi Toro, Emanuela Moscardini, Ludovica M. Baldassari, Flavia Forte, Jacopo Coletta, Emma Palo, Vittoria Cosentino, Fabio Angelini, Alba Arratibel Plazaola, Francesca Pagnanelli, Pietro Altimari

**Affiliations:** 1 Eco Recycling Srl, Via Francesco Siacci, 4, 00197 Rome, Italy; luigi.toro@ecorecycling.eu (L.T.); emanuela.moscardini@ecorecycling.eu (E.M.); ludovica.baldassari@ecorecycling.eu (L.M.B.); flavia.forte@ecorecycling.eu (F.F.); 2 KT—Kinetics Technology Spa, Viale Castello della Magliana, 27, 00148 Rome, Italy; e.palo@kt-met.it (E.P.); v.cosentino@kt-met.it (V.C.); f.angelini@kt-met.it (F.A.); 3 Membrane Technology and Process Intensification/Materials and Processes, TECNALIA, Basque Research and Technology Alliance (BRTA), Mikeletegi 2, 20009 San Sebastian, Spain; alba.arratibel@tecnalia.com; 4 Department of Chemistry, Sapienza University of Rome, P.Le A. Moro 5, 00185 Rome, Italy; francesca.pagnanelli@uniroma1.it (F.P.); pietro.altimari@uniroma1.it (P.A.)

**Keywords:** recycling, ceramic membranes, palladium, CCS technology, hydrogen, integrated CO_2_ capture, economic analysis, circular economy, prototype plant

## Abstract

The aim of the present work is the recycling treatment of tubular α-Al_2_O_3_-supported ceramic membranes with a Pd/Ag selective layer, employed in hydrogen production with integrated CO_2_ capture. A nitric acid leaching treatment was investigated, and recovered ceramic supports were characterized, demonstrating their suitability for the production of novel efficient membranes. The main objective was the metal dissolution that preserved the support integrity in order to allow the recovered membrane to be suitable for a new deposition of the selective layer. The conditions that obtained a satisfactory dissolution rate of the Pd/Ag layer while avoiding the support to be damaged are as follows: nitric acid 3 M, 60 °C and 3.5 h of reaction time. The efficiency of the recovered supports was determined by nitrogen permeance and surface roughness analysis, and the economic figures were analysed to evaluate the convenience of the regeneration process and the advantage of a recycled membrane over a new membrane. The experimentation carried out demonstrates the proposed process feasibility both in terms of recycling and economic results.

## 1. Introduction

Carbon dioxide is one of the major products of combustion with carbon-containing fuels.

In 2020, more than 80% of EU (European Union) primary energy was based on fossil and nuclear sources. Oil and petroleum products held the biggest share (34.5%), followed by natural gas (23.7%), whereas solid fossil fuels represented 10.2% [1]. These also represent a fundamental resource for many key industrial sectors, such as the chemical and metallurgical industries, where they are not easily replaceable by alternative material with a lower environmental impact.

The highest amount of CO_2_ emissions come from the burning of fossil fuels for energy and cement production (about 34.81 billion t in 2020 have been released [2]), but also for transportation, industry processes, and commercial, residential and agricultural applications [3].

Such emissions cause an increase in CO_2_ concentration in the atmosphere and contribute (~80%) to all greenhouse gases (GHG) [4], causing higher Earth surface temperature, global warming and other severe climatic disturbances. 

In the past years, a great deal of effort in modern low-emission energy technologies was directed at activities leading to decreased gaseous pollutant emissions. Carbon capture utilization and storage (CCUS) is a family of methods to reduce the emission of CO_2_ from fossil-fuelled power plants [5,6]. This technique represents a promising option to deal with unavoidable CO_2_ emissions from fossil fuels with the need to reduce greenhouse gas emissions [4].

Therefore, carbon capture and storage (CCS) is a method for capturing the concentrated CO_2_ in flue gas from fossil-fuelled power plants and for storing them in a specific area. There are three methods of CO_2_ capture: pre-combustion carbon capture, post-combustion carbon capture, and the oxy-combustion carbon capture method [5].

Among the different gas separation methods, membrane technology has been investigated for removing CO_2_ from mixtures with light gases such as CH_4_, N_2_ and H_2_, and optimal membranes with high CO_2_ permeability and high CO_2_/light gas selectivity are of great interest [7]. Membrane processes are gaining a larger acceptance in the industry and market and are competing with consolidated operations such as pressure swing absorption and cryogenic distillation. The key for new applications of membranes in challenging and harsh environments is the development of new tough, high-performance materials [8]. 

Furthermore, this technology should ensure proper gas separation properties such as permeability and selectivity. There are three types of membrane materials: polymeric membranes (organic), ceramic membranes (inorganic), and hybrid ones [9].

The membranes modules are high added-value devices, and their cost depends on both the support and the type of selective layer used. In the case of H_2_ selective membranes, for example, the cost can even reach 17 kEUR/m^2^ due to the cost of the Pd, the selective layer, and the cost of the support (today, it varies between 1000 and 4000 EUR/m^2^). Their recyclability is a determinant aspect that allows the membranes to be attractive to the market, both to reduce the unit cost of production by recovering its constituent elements and for the environment itself. The regeneration process is required when the membranes reach the end of life or when their chemical and mechanical properties are no longer able to guarantee compliance with the specifications in terms of selectivity and permeability.

In the present work, a recycling of the damaged Pd/Ag-based membranes has been carried out. The most challenging aspect of the presented activity was achieving the almost complete dissolution of the Pd/Ag, avoiding damage to the ceramic support from a physical, chemical and mechanical point of view in order to allow the tubular membranes to be suitable for a new deposition of the selective layer. The suitability of the recovered modules was verified through permeability and mechanical tests.

The economic figures were analysed to evaluate the convenience of the regeneration process by comparing the cost of a recycled membrane with the cost of a new one. The production cost of the recycled membrane was evaluated as the sum of OPEX and CAPEX for the recycling process and then by adding the cost of re-deposition of the selective layer. The cost of the new membranes was calculated on the basis of Tecnalia’s experience.

The membranes used in the presented work were produced by Tecnalia in the frame of the EU H2020 project “MEMBER”. These membranes were employed in a prototype plant (MA-SER plant, Membrane Assisted Sorption Enhanced Reforming plant) for pure hydrogen production with integrated CO_2_ capture at the facilities of IFE-HyNor. 

In the open literature, there are some studies concerning the recovery of palladium and silver from different types of industrial wastes via a hydrometallurgical process. 

In general, these wastes are previously mechanically treated in order to obtain a sample with an adequate particle size to allow the dissolution of target metals to be more efficient. Furthermore, these wastes are usually treated with mineral acid as nitric, hydrochloric and their mixture with hydrogen peroxide [10,11,12,13].

Studies in which less conventional mixtures are used are also reported in the literature [14,15,16].

The “destructive approach” for preparing the samples related to the studies above is adequate since the primary objective for such treatments consists of the recovery of precious metals.

However, membrane recycling should involve both the metal dissolution and the undamaged support recovery. The membranes’ support (in particular when a metallic support is used) often represents the more valuable constituent of the membranes’ modules. The treatment of the entire tubular membranes makes the recovery process a challenging one because of the disadvantageous fluid dynamics behaviour and the smaller contact surface between the acid mixture and the metal particles.

The open literature refers to only a few studies on the recycling process of the entire tubular membranes [17,18]. In comparison to the cited references, the proposed process was developed with the aim of defining a specific recycling treatment for the supported Pd/Ag-based membranes with particular attention on the identification of the operating conditions and the reagents to allow the recycling treatment to be economically feasible in an industrial-scale plant.

Eco Recycling and KT developed a specific method for the treatment of exhaust Pd-based membranes used for H_2_ separation in the frame of a regional project. The project aim was the development and optimization of a hydrometallurgical process for the recovery of both the support and the Pd/Ag dissolution [19]. 

The research investigated mineral reagents, inorganic salts and organic compounds. However, non-conventional leaching mixtures were shown to be ineffective for the recycling of entire membrane modules. Among the mineral acids tested, the nitric acid resulted to be the most efficient reagent for the target metal dissolution rate. Furthermore, its usage involves a lower environmental impact than other mineral ones (e.g., hydrochloric acid and hydrogen peroxide mixtures), making it more adequate for its application on an industrial scale.

The process developed on the laboratory scale was then tested in a pilot plant at Eco Recycling’s industrial site in Civita Castellana (VT, Italy). The process was developed considering the operating conditions applicable on the prototype scale. The pilot plant, already owned by Eco Recycling, was designed specifically for the treatment of tubular membranes for hydrogen purification. The reactor was the result of a fluid dynamic study specific to maximize the yield of the recycling process.

## 2. Materials and Methods

### 2.1. Recycling Treatment

The recycling treatment was focused on the selective dissolution of the exhaust membrane’s external coating by preserving the ceramic support integrity. During the operating life of the membranes, the metal coating is damaged with an unacceptable loss of selectivity towards hydrogen. The recycling treatment consists of regenerating a suitable support for a new metal deposition in order to obtain a new performing membrane module.

#### 2.1.1. Samples Main Characteristics

The experimentation was performed on two different ceramic-supported membranes (MEMBR1 and MEMBR2) produced under the same recipe by Tecnalia in two different production batches. The tubular membranes consist of a metallic coating composed of a specific palladium and silver alloy, containing about 4–5% silver deposited onto an α-alumina porous support as reported previously [20], which lends the module the required mechanical strength. Further details are reported in Table 1.

Furthermore, additional experiments were performed on a metallic-supported tubular membrane (MEMBR3) composed of a Pd/Ag coating deposited by electroless plating technique onto a Hastelloy-X porous support modified with an α-Al_2_O_3_/YSZ interdiffusion layer as reported previously [21]. 

The membranes were cut to obtain a suitable number of small samples to test the planned process conditions during experimentation. The small samples were identified with an identification code. For example, M1#5A is referred to as MEMBR1 (M1-), test number (5), with sample identification between the two samples treated in a parallel test (-A or -B). Generally, parallel tests were carried out under the same process conditions in order to further validate the obtained results. Furthermore, a larger sample was also obtained from both the membranes (M1 from MEMBR1 and M2 from MEMBR2) in order to test it under the best process conditions established during the experimentation on the small samples. The two recovered samples were then tested by Tecnalia to verify their suitability for a new Pd/Ag coating deposition. The nitrogen permeance and surface roughness (R_a_ and R_t_) measurements were performed in order to validate the recovered supports. The roughness was analysed with a Vecco Dektak 150 contact profilometer with a 2 µm radius stylus tip. Five measurements of 4 mm each were performed per sample at different locations along the treated tubes. Ra is the arithmetical mean roughness, and Rt is the total height of the roughness profile, the difference between the highest peak to valley distance.

**Figure 1:**
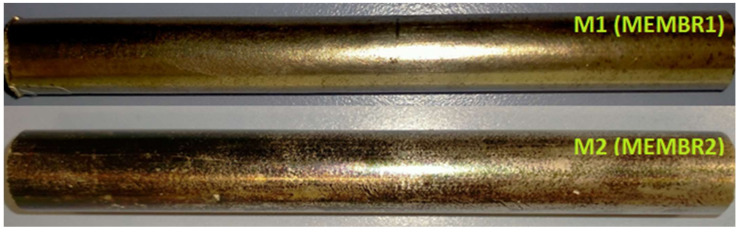
Samples M1 (**above**) and M2 (**below**) before the recycling treatment.

The small samples were cut from MEMBR1 and treated during the leaching tests (M1#1 M1#2, M1#3A, M1#4A, M1#5A, M1#6A and M1#3B, M1#4B, M1#5B, M1#6B). Two samples were cut from MEMBR2 and treated during the leaching tests (M2#1A and M2#1B). Two large samples (M1 and M2 shown in Figure 1) were cut from both the membranes and treated to dissolve the coating with the most efficient leaching conditions previously determined for the smallest samples. The weight and size of each sample was determined by using a laboratory analytical balance and a callipers tool. 

#### 2.1.2. Leaching Treatment

The objective of the experimentation was the optimization of the process conditions (acid concentration, temperature and reaction time). 

The parameter which allowed for us to assess the efficiency of the recycling treatment was the palladium dissolution yield. It is both the most noble and abundant metal in the coating alloy and the more resistant component to the leaching solution. Extraction yields were determined comparing the leached palladium with the total content of Pd in each sample. Another parameter which was considered to assess the success of the test was the aluminium content in the leach liquors: a low amount of this component in the solution indicated a low solubilisation of alumina-based support.

For the leaching step, nitric acid (65% wt., Carlo Erba) was used. The experiments were performed using jacketed glass reactors and a thermostatic water bath for temperature control. A magnetic stirrer was arranged to keep the solution properly mixed (300 rpm). Regarding the L/S ratio, it was chosen to adopt the same value used on the prototype plant. The extra volume of liquid results from the prototype geometry which was designed to fulfil the fluid-dynamic requirements which can maximize the extraction yields. The acid-based solution can be recycled to the next leaching step and can be reused at least 10 times before discharging it. 

Small solution samples were taken during the treatments to determine the dissolution rate over time. In order to compensate for the palladium removed during the samplings, adequate corrections were carried out in the yields’ calculations. All the analyses were performed using an atomic absorption spectrophotometer (AAS-ANALYTIKJENA contrAA 300).

The starter process conditions were set based on the Eco Recycling’s method. The planned leaching experiments were finalized to test different specific conditions of temperature, concentration and reaction time. In particular, the tests were performed at 50 and 60 °C, with a nitric acid concentration of 3 and 4 M and a maximum treatment time of 7 h. The specific leaching conditions for small samples are reported in Table 2. 

The larger samples, M1 and M2, were tested under the best conditions resulting from the previous experimentation performed on the small samples. The L/S ratio adopted for these tests was decreased compared to the ratio used for small samples, but it was still in stoichiometric excess. It was a reasonable choice due to the leaching agent excess and the size of the samples. The sample characteristics and treatment conditions are shown in Table 3. 

**Figure 2:**
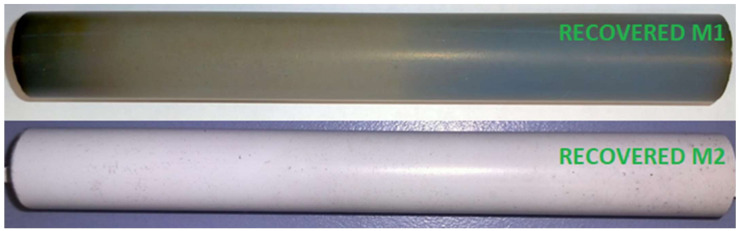
Samples M1 (**above**) and M2 (**below**) after the recycling treatment.

A washing treatment was performed after the leaching in order to remove the solution residues which could crystallize and collapse inside the porous structure. The M1 and M2 post-leaching residues were washed in water (325 mL) at 60 °C for 1 h under continuous stirring (300 rpm). The recovered membranes after the leaching and the washing treatments are shown in Figure 2.

### 2.2. Solid Residues Treatment

The solid residues treatment tests were necessary in order to quantify the total content of Pd and Ag in each small sample and to estimate the yields of the leaching tests. For the solid residue treatment tests, aqua regia was prepared with nitric acid (65% wt., Carlo Erba) and hydrochloric acid (37% wt., Carlo Erba). The laboratory setup was the same as already described for the leaching treatment in Section 2.1.

Each solid residue after the leaching treatment was further treated to dissolve any Pd/Ag residue. This can establish the Pd/Ag content for the overall MEMBR1 and MEMBR2 and independently define the dissolution yield of the process for each sample. Parallel tests were carried out under the same solid residue treatment conditions in order to validate the obtained results. The experimental conditions are reported in Table 4.

Solid residue treatment tests in aqua regia were carried out only on the small samples, as the longer ones, M1 and M2, were characterized at Tecnalia to verify their suitability for a new Pd/Ag coating deposition.

## 3. Results and Discussion

### 3.1. Leaching Results

The leaching results expressed as extracted mg of metal per gram of treated sample are reported in Table 5 and Table 6. The palladium extraction yields over time are instead reported in Section 3.3.

M1 was treated under the same process conditions of the small samples M1#4A and M1#4B. However, the palladium and silver extracted during leaching was slightly inferior to the related small samples. M2 was treated under the same process conditions of M2#1A and M2#1B. The results as extracted metals are in line with the related small samples. The M1#1 lye was analysed to evaluate the potential aluminium content. If was found that no aluminium dissolved in the solution (Al was not detectable in leach liquor from leaching tests since it was <<0.5 ppm in undiluted solution). This was considered a sign regarding the chemical stability of the α-alumina support against the nitric acid-based solution. 

### 3.2. Solid Residues Treatment Results

The solid residue treatment results, expressed as extracted milligram of palladium per gram of treated sample, are reported in Table 7. Palladium was used as the main reference for the extraction yield calculation since it is the most abundant and noble metal in the coating alloy.

The low quantities of palladium found in these tests suggested that the palladium was almost completely dissolved in the previous leaching treatment. Regarding the silver, its total absence in the undiluted solution samples was verified.

In conclusion, considering the sum of the dissolved metals in the leaching test (Table 5) and in the solid residue treatment (Table 7), the total Pd/Ag content in the small samples is reported in Table 8, including the silver percentage in the alloy.

The palladium and silver content in the samples are consistent: the slight differences in metal content found for the small samples are due to the samples’ heterogeneity. The weighted mean of the results obtained for each sample was calculated. In this way, an overall coating composition for MEMBR1 and MEMBR2 was obtained, including the overall Ag percentage in the alloy (Table 9).

The palladium content in MEMBR1 and MEMBR2 is similar. However, the silver content in MEMBR2 is slightly lower than the one found for MEMBR1. Accordingly, the alloy shows a lower silver percentage in MEMBR2 than in MEMBR1. In order to have an even strong indication of the chemical stability of the support in nitric acid-based environments, the aluminium was analysed to quantify its dissolution (Table 10).

Despite the harsh conditions tested (aqua regia), the dissolution of aluminium can be considered negligible. It can be concluded that the nitric acid leaching process does not dissolve the α-alumina support.

### 3.3. Effect of Operative Conditions on Leaching Yields

In order to evaluate the progress of the leaching process over time and to better compare the tests result, yield vs. time curves were developed for the palladium dissolution, since they represent the target metal for the evaluation of the process conditions’ effectiveness.

**Figure 3:**
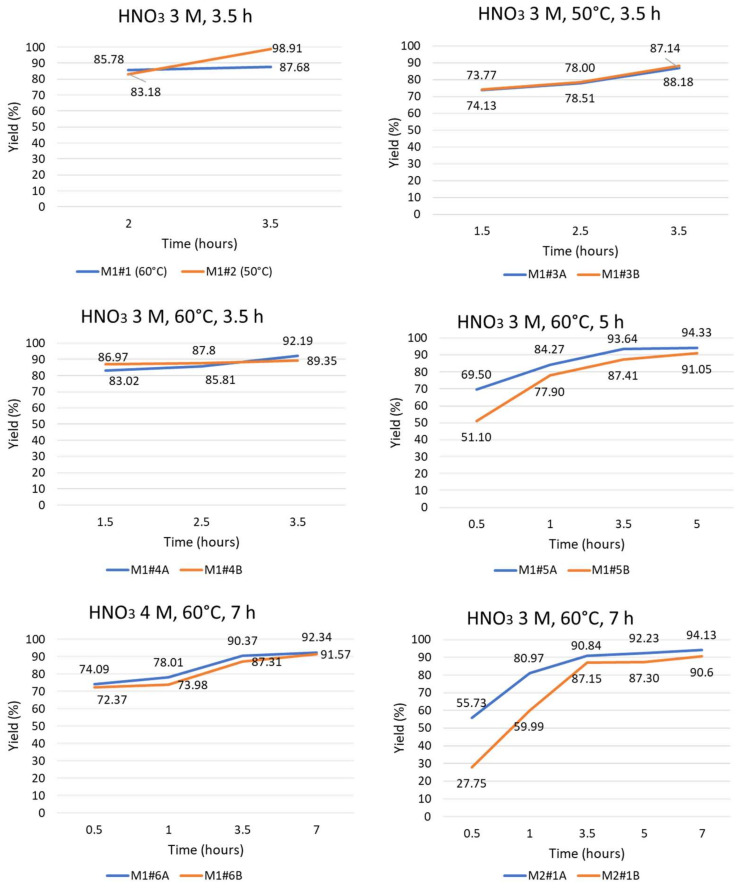
Yields vs. time curves.

Small solution samples were taken at defined time intervals during the leaching tests. The number of taken samples was defined progressively and in accordance with the first dissolution results obtained. These latter ones showed a fast dissolution rate in the initial phase of the reaction. For this reason, additional samplings were made in the initial stages of the leaching tests. Furthermore, the duration of the tests also varied during experimentation. Based on the results given by the first tests, it was considered interesting to investigate the leaching behaviour for longer reaction times. Therefore, the leaching yields for palladium were calculated at each sampling time by comparing the leaching results with the total content of Pd in the samples (obtained by adding the metals extracted with the leaching and those derived from the solid residue treatment). The silver extraction was assumed to be complete in every tested condition. The results are shown in the graphs of Figure 3. Regarding the M1 and M2 sample, since the solid residue treatment tests were not carried out, the results of the samplings over time are reported as mg/g.

Leaching of palladium is initially a fast reaction, but a plateau in dissolution yield is reached after about 3.5 h (which corresponds to about 90% of the dissolution yield). There were not significant variations found in terms of time and yield at the plateau in the different experimental conditions. The maximum obtained yield was about 95%. Furthermore, small differences were found between the parallel test behaviours. They can be due to the heterogeneity of the samples, which is reasonable considering the treatment of end-of-life membranes. The overall results, however, are consistent. In Table 11, the final yields in every leaching experiment are summarized.

In Table 12, the metals extracted over time for M1 and M2 samples are reported.

The leaching trend of M1 is comparable to the results of tests M1#4A, M1#4B, M1#5A and M1#5B, which were carried out under the same conditions. A fast dissolution of Pd was initially observed, and then a plateau was reached. The leaching trend of M2 is comparable to the results of the tests M2#1A and M2#1B, which were carried out under the same conditions. A fast initial peak and a subsequent slowdown and plateau for the Pd dissolution rate were observed. 

### 3.4. Characterization of Treated Samples

The recovered porous tubes have to offer enough surface properties (low roughness) and enough gas transport properties (nitrogen permeance) to be employed again as a support for Pd-Ag membrane deposition. The nitrogen permeance of the recovered tubes was evaluated at room temperature at a pressure difference of 0.3 barg. In Table 13, the permeation properties and roughness of the treated tubes and a fresh porous α-Al_2_O_3_ tube are shown. The measured surface roughness of the treated samples (M1 and M2) are below the fresh α-Al_2_O_3_ tube; hence, M1 and M2 have good surface qualities for the deposition of a Pd-Ag layer on top of it. Complete validation of the samples is also based on the nitrogen permeation properties, which are above 1·10^−5^ mol m^−2^ s^−1^ Pa^−1^ for both cases. Therefore, the recovered tubes can be reused as support for deposition of the Pd-based membrane.

### 3.5. Process Transferability on Metal-Supported Membranes

The optimization of the leaching mixture and the operating conditions, could also validate the recycling process for the exhaust metallic-supported membranes. In addition, these membranes are tubular ones, but their support is made of a metallic alloy in Hastelloy X, with an intermetallic barrier of α-Al_2_O_3_/YSZ and a selective layer of Pd/Ag with a silver content of about 4–5%. 

The same leaching parameters employed for the ceramic-based membranes were implemented to treat the metallic-based membranes. The palladium extraction yield was about 97%, with the following leaching operative conditions:

- Nitric acid concentration: 4 M;
- Temperature: 60 °C;
- Reaction time: 4.5 h;

Table 14 shows the results of the preliminary leaching tests of the metallic-supported membranes:

These preliminary tests will be further validated and improved upon with new experimentation, but the results show good efficiency of the conditions tested towards palladium solubilisation.

## 4. Prototype Plant and Process Scale-Up

The process developed on the laboratory scale was then tested on a larger scale to validate the recycling process. The pilot plant was built as part of the “HYRPAM” project and was revamped during the “MEMBER” project for the specific treatment of the membrane modules manufactured in this last project and previously described in this work. The prototype is located at Eco Recycling’s operative headquarters in Civita Castellana (VT, Italy). 

The plant consisted mainly of two interconnected units: a tank where the mixing and heating of the acid solution occurred and a specific reactor where the leaching treatment took place. The reactor was specifically designed in order to produce a specific fluid dynamic inside the reaction area that maximizes the efficiency of leaching. Both the mixer and the reactor were provided with an integrated probe to allow for the online monitoring of temperature, pH and redox potential. The equipment and the pipeline were design in order to be chemically stable towards the leaching mean. The storage tanks area was set to allow for the storage of the acid solution, the cleaning water and the collected wastewater. In order to limit the emissions into the atmosphere and to comply with local regulations, a specific vapour abatement system was designed and built. 

**Figure 4:**
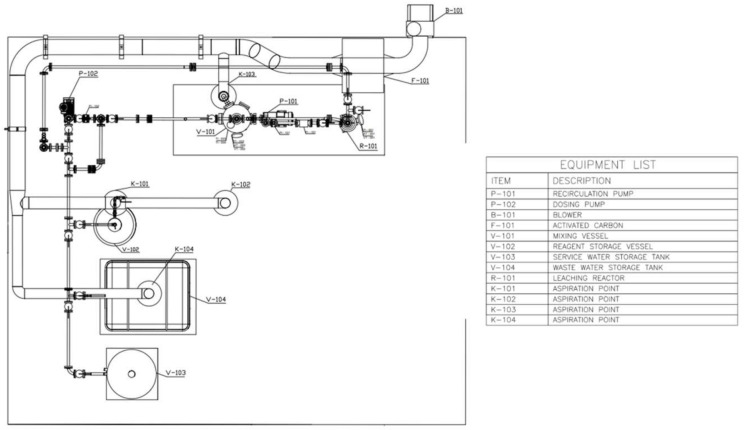
Prototype plant for Pd-based membrane recycling.

The plant allows for the treatment of at most eight 25 cm long membranes per batch. In Figure 4, the plant layout is shown.

## 5. Economic Analysis

The preliminary economic evaluation carried out was based on the comparison between the cost/m^2^ of a new membrane compared to one treated for reuse. 

The cost of the new membrane was calculated on the basis of Tecnalia’s experience. For a Pd-based membrane on ceramic support, the total cost is around 17 kEUR/m^2^, where 70% of the cost (about 12 kEUR/m^2^) is only the cost of the selective Pd layer.

For the recycled membranes, both fixed costs and variable costs were taken into consideration. 

The fixed costs were assessed based on the process scale up and layout starting from the estimate of the equipment, piping, engineering, and civil works and by considering a depreciation of 10% over 15 years. This leads to a total capital investment of 35 EUR/m^2^. The variable costs were assessed based on the consumption per m^2^ of the pilot unit, which was assumed to treat eight membrane modules together. To these were then added the cost of direct personnel considering two operators and an administrative cost of 20%. The final variable cost is therefore of around 2 kEUR/m^2^, and by adding the fixed costs, a final production cost of 2.1 kEUR/m^2^ is obtained. The possibility to recycle the leaching solution was also considered. Based on Eco Recycling expertise, it could be possible to reuse the acidic solution at least 10 times (even if more tests are needed to confirm these data). In this case, the variable cost is 1.2 kEUR/m^2^, and the total production cost becomes 1.3 kEUR/m^2^.

After having estimated the cost of recycling the support, the final cost of the membrane was evaluated while adding the cost of the re-deposition of the selective layer. The latter was assumed to be equal to the cost of a new Pd layer, thus equaling 12 kEUR/m^2^, which, as seen above, has the greatest weight on the total cost of the membrane. The final cost of a recycled membrane therefore becomes about 15 kEUR/m^2^. In Table 15, the breakdown costs of a recycled ceramic Pd-based membrane are reported. 

In Table 16, the comparison between a new and recycled ceramic Pd-based membrane is reported.

**Figure 5:**
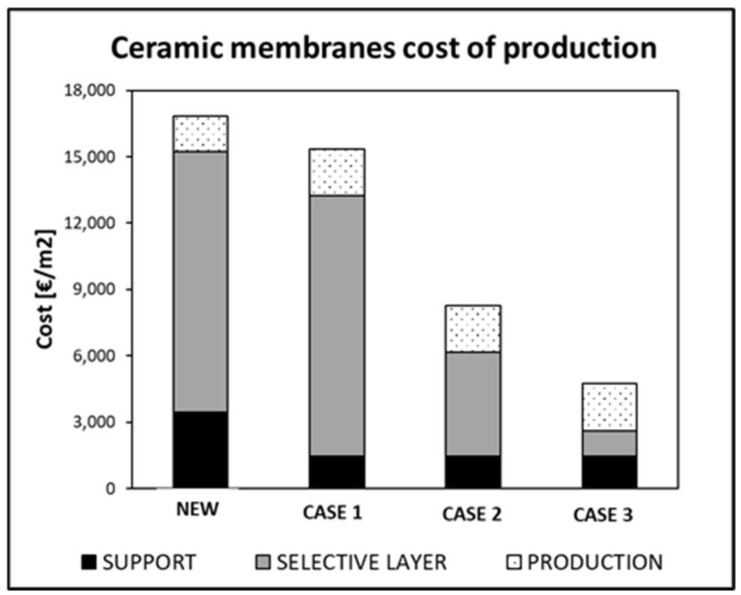
Breakdown cost for ceramic Pd membranes, assuming support recycle (case 1), support recycle + 60% selective layer recovery (case 2), and support recycle + 90% selective layer recovery (case 3).

The final cost of the membrane is therefore lower than the new membrane. To reduce this cost even more, the recovery of the selective layer can be investigated. In Figure 5, the cost of a new ceramic membrane is compared with that of recycled membranes, assuming only to recycle the support (case 1) and to recover the selective layer (60% recovered for case 2, 90% recovered for case 3).

It is evident how the total costs can decrease up to 70% of the initial value due to the recovery of the palladium. Therefore, as an outlook, further research should be carried out on this aspect.

## 6. Conclusions

The present work focused on the recycling of exhaust Pd/Ag membranes with a ceramic support. These membranes were produced within the frame of the H2020 project “MEMBER” and were tested in a pilot plant for selective hydrogen purification with integrated CO_2_ capture.

The proposed recovery process was improved to be efficient under economical operating conditions. In order to check the effectiveness of the recycling process, the extraction yields of palladium were chosen as the target indicator during the leaching experimentation. In addition, silver extraction was evaluated.

The conditions that allowed us to obtain a satisfactory dissolution rate of the Pd/Ag coating avoiding the support to be damaged by the acidic solution are as follows:

- Leaching agent: nitric acid 3 M;
- Temperature: 60 °C;
- Reaction time: 3.5 h;

Longer reaction times do not lead to a substantial increase in the solubilisation of palladium. Furthermore, despite the residual palladium traces on the membrane surface, the recovered ceramic support was demonstrated to be suitable for a successive deposition of the metal coating and therefore the production of new efficient membranes.

The economic analysis showed a 9% reduction in production cost compared to a new membrane, only considering the recycle of the support. A further reduction could be achieved by also recovering the selective layer, which currently represents the heaviest contribution for both new and recycled membranes.

## Figures and Tables

**Table 1:** MEMBR1 and MEMBR2 characteristics.

Parameter	MEMBR1	MEMBR2
Ceramic support	α-Al_2_O_3_	α-Al_2_O_3_
Pore size	100 nm	100 nm
Interdiffusion barrier	No	No
Selective layer	Pd/Ag (~4–5%wt Ag)	Pd/Ag (~4–5%wt Ag)
Thickness selective layer	4–5 μm	4–5 μm
Overall length	22 cm	25.5 cm
Outer diameter	14.6 mm	14.3 mm

**Table 2:** Tested leaching conditions on small samples.

SAMPLE	Size (d × h, mm)	Weight (g)	HNO_3_ (M)	Temperature (°C)	Time (h)
M1#2	1.46 × 0.32	1.029	3	50	3.5
M1#3A	1.46 × 0.92	2.848			
M1#3B	1.46 × 0.86	2.760			
M1#1	1.46 × 0.31	1.018	3	60	3.5
M1#4A	1.46 × 0.88	2.693			
M1#4B	1.46 × 0.92	2.758			
M1#5A	1.46 × 0.93	3.049	3	60	5
M1#5B	1.46 × 0.90	2.767			
M1#6A	1.46 × 0.88	2.556	4	60	7
M1#6B	1.46 × 0.88	2.545			
M2#1A	1.43 × 0.90	2.882	3	60	7
M2#1B	1.43 × 0.90	2.851			

**Table 3:** M1 and M2 characteristics and tested leaching conditions.

**SAMPLE M1**	
Length (cm)	11.4
Weight (g)	37.54
**SAMPLE M2**	
Length (cm)	11.8
Weight (g)	38.57
**Leaching Conditions**	
Reagent	HNO_3_ (3 M)
Temperature (°C)	60 °C
Solution volume (mL)	M1: 1036 M2: 1072
Stirrer speed (rpm)	300

**Table 4:** Solid residue treatment conditions for small samples.

Parameter	Type/Value
Reagent	Aqua regia
Temperature (°C)	60
Solution Volume (mL)	50
Test duration (h)	3

**Table 5:** Leaching tests results for small samples.

SAMPLE	Pd (mg/g)	Ag (mg/g)
M1#1	2.98	0.21
M1#2	3.26	0.16
M1#3A	2.80	0.18
M1#3B	2.90	0.19
M1#4A	3.01	0.19
M1#4B	2.93	0.17
M1#5A	3.28	0.19
M1#5B	3.23	0.16
M1#6A	3.04	0.19
M1#6B	3.13	0.22
M2#1A	2.92	0.09
M2#1B	2.79	0.15

**Table 6:** M1 and M2 leaching test results.

SAMPLE	Pd (mg/g)	Ag (mg/g)
M1	2.67	0.14
M2	2.87	0.12

**Table 7:** Solid residue treatment results for small samples.

SAMPLE	Reagent	Pd (mg/g)
M1#1	Aqua regia	0.42
M1#2	Aqua regia	0.04
M1#3A	Aqua regia	0.41
M1#3B	Aqua regia	0.39
M1#4A	Aqua regia	0.26
M1#4B	Aqua regia	0.35
M1#5A	Aqua regia	0.20
M1#5B	Aqua regia	0.32
M1#6A	Aqua regia	0.25
M1#6B	Aqua regia	0.29
M2#1A	Aqua regia	0.18
M2#1B	Aqua regia	0.29

**Table 8:** Palladium and silver content in small samples.

SAMPLE	Pd (mg/g)	Ag (mg/g)	Alloy (% Ag)
M1#1	3.39	0.21	5.77
M1#2	3.29	0.16	4.69
M1#3A	3.22	0.18	5.29
M1#3B	3.29	0.19	5.41
M1#4A	3.27	0.19	5.51
M1#4B	3.28	0.17	4.87
M1#5A	3.48	0.19	5.12
M1#5B	3.54	0.16	4.21
M1#6A	3.29	0.19	5.53
M1#6B	3.42	0.22	6.03
M2#1A	3.10	0.09	2.89
M2#1B	3.08	0.15	4.66

**Table 9:** MEMBR1 and MEMBR2 metal content.

SAMPLE	Pd (mg/g)	Ag (mg/g)	Alloy (% Ag)
MEMBR1	3.35	0.18	5.23
MEMBR2	3.09	0.12	3.78

**Table 10:** Results of aluminium support dissolution in solid residue treatment.

SAMPLE	Method	Aluminium Dissolution (mg/g)
M1#1	Aqua regia, 60 °C, 3 h	0.12

**Table 11:** Leaching yields in leaching tests.

SAMPLE	HNO_3_ (M)	Temperature (°C)	Time (h)	Pd Yield (%)
M1#2	3	50	3.5	98.91
M1#3A				87.14
M1#3B				88.18
M1#1	3	60	3.5	87.68
M1#4A				92.19
M1#4B				89.35
M1#5A	3	60	5	94.33
M1#5B				91.05
M1#6A	4	60	7	92.34
M1#6B				91.57
M2#1A	3	60	7	94.1
M2#1B				90.6

**Table 12:** M1 and M2 leaching over time.

**SAMPLE M1**		
Time	Pd (mg/g)	Ag (mg/g)
0.5 h	1.44	0.09
1 h	2.13	0.12
3.5 h	2.67	0.14
**SAMPLE M2**		
Time	Pd (mg/g)	Ag (mg/g)
0.5 h	1.20	0.07
1 h	2.53	0.11
3.5 h	2.87	0.12

**Table 13:** Surface roughness and nitrogen permeance of treated ceramic-supported membranes.

SAMPLE	Ra (µm)	Rt (µm)	N2 Permeance (mol m^−2^ s^−1^ Pa^−1^)
M1	0.35 ± 0.04	3.39 ± 1.41	1.29·10^−5^
M2	0.31 ± 0.02	2.83 ± 1.49	1.49·10^−5^
α-Al_2_O_3_ *	0.52 ± 0.12	6.49 ± 2.75	>1·10^−5^

* Fresh alumina porous support.

**Table 14:** Leaching yields of the metallic-based membrane (MEMBR3).

SAMPLE	HNO_3_ (M)	Temperature (°C)	Time (h)	Pd Yield (%)
M3#1A	4	60	4.5	97.72
M3#1B				97.52
M3#2A	4	60	4.5	97.29
M3#2B				96.52
M3#3A	3.5	60	7	97.73
M3#3B				97.67
M3#4A	3	60	7	97.15
M3#4B				92.43

**Table 15:** Breakdown costs of recycled ceramic Pd-based membrane.

	% Cost (EUR/m^2^)
OPEX	15.0%
CAPEX	0.3%
Re-deposition selective layer	84.7%

**Table 16:** Comparison between new and recycled ceramic Pd-based membrane.

	Cost of Production (kEUR/m^2^)	% of Reduction
New ceramic Pd-based membrane	17.0	
Recycled ceramic Pd-based membrane	15.0	9.0%

## Data Availability

Not applicable.
